# Safety of concurrent treatment of dogs with fluralaner (Bravecto™) and milbemycin oxime - praziquantel

**DOI:** 10.1186/s13071-014-0481-y

**Published:** 2014-10-15

**Authors:** Feli M Walther, Petr Fisara, Mark J Allan, Rainer KA Roepke, Martin C Nuernberger

**Affiliations:** MSD Animal Health Innovation GmbH, Zur Propstei, 55270 Schwabenheim, Germany; MSD Animal Health, 26 Artisan Road, Seven Hills, NSW 2172 Australia

**Keywords:** Bravecto™, Fluralaner, Dog, Safety, Milbemycin oxime, Praziquantel

## Abstract

**Background:**

Fluralaner (Bravecto™; Merck/MSD Animal Health) is a novel systemic ectoparasiticide for dogs providing long-acting flea and tick control after a single oral dose. Milbemycin oxime and praziquantel are routinely used to control *Dirofilaria immitis* and intestinal worm infections in dogs. The safety of concurrent use of fluralaner and a commercially available milbemycin oxime plus praziquantel combination tablet, in particular with regard to gastrointestinal symptoms, was investigated using oral doses at or above the maximum recommended rates.

**Findings:**

Some minor and transient clinical findings were observed during the study period; however, none of these was considered to be related to concurrent treatment with fluralaner and milbemycin oxime plus praziquantel, or to the use of either product alone.

**Conclusions:**

Concurrent treatment with fluralaner, milbemycin oxime and praziquantel is well tolerated in dogs.

## Findings

Fluralaner (Bravecto™; Merck/MSD Animal Health) is a systemically administered insecticidal and acaricidal product. Numerous studies including a recent field study in dogs have shown that a single fluralaner dose administered orally as chewable tablet provides flea and tick control for twelve weeks [1].

Milbemycin oxime is active against larval and adult stages of intestinal nematodes as well as against larval blood stages of heartworm (*Dirofilaria immitis*). Praziquantel is active against cestodes and trematodes [2,3].

Dogs may concurrently be exposed to tick and flea infestations, heartworm infestations and intestinal worm infestations, therefore veterinarians may choose to administer fluralaner concurrently with milbemycin oxime and praziquantel. For both products mild and transient gastrointestinal effects, like vomiting, inappetence, drooling and diarrhea, may occur after oral administration [2–4]. To confirm the safety of the concurrent use of fluralaner and milbemycin oxime plus praziquantel, in particular with regard to gastrointestinal symptoms, a study was conducted in healthy dogs. Fluralaner tablets (Bravecto™) and a commercially available milbemycin oxime plus praziquantel combination tablet were orally administered at or above the recommended treatment dose (recommended treatment dose: 25–56 mg/kg BW for fluralaner, 0.5 - 5 mg/kg BW for milbemycin oxime, 5–50 mg/kg BW for praziquantel) [2–4].

## Methods

The study was conducted in Queensland, Australia, with the authorization of relevant regulatory authorities (Queensland Department of Agriculture, Fisheries and Forestry, approval no. CA 2014/05/768).

Twenty healthy male and female dogs of various breeds, 1.4 – 8.6 (mean 5.3) years of age and weighing 5.8 – 33.9 (mean 21.6) kg, were randomly assigned to two study groups. Dogs were acclimatized for 7 days before treatment. On day 0, dogs of the treatment group were administered fluralaner chewable tablets and commercially available milbemycin oxime plus praziquantel combination tablets. Dogs of the control group received the milbemycin oxime plus praziquantel combination tablets only.

The actual doses administered to dogs of both study groups are presented in Table 1. Dogs in both groups were fed directly before treatment as recommended on the product leaflets [2–4].

**Table 1:** **Doses of fluralaner, milbemycin oxime and praziquantel administered to dogs of the treatment and control group**

**Active ingredient (mg/kg)**	**Treatment group**		**Control group**	
	**Dose range**	**Mean dose**	**Dose range**	**Mean dose**
**Fluralaner**	50 – 87	64	-	-
**Milbemycin oxime**	2.6 – 4.4	2.9	2.5 – 4.1	2.8
**Praziquantel**	26 – 44	29	25 – 41	28

Dogs of both groups were observed for general health during the first hour following treatment and were examined by a veterinarian at 2, 3, 4, 5, 6, 9, 12, 24, 36, 48, 60, 72 and 84 hours, and 4, 5, 6 and 7 days after treatment. The veterinarian examined for abnormalities in behavior, locomotion, coat and skin, respiration, eyes, ears, nose, oral cavity, mucous membranes, capillary refill time, pulse palpation, vomitus, feces and urine as present in pen, and any other visible abnormalities.

Veterinary examinations continued on study days 16 and 28 (examinations included assessment of abnormalities in behavior, locomotion, auscultation of heart and thorax, heart rate, respiratory rate, pulse palpation, mucous membranes, capillary refill time, abdominal palpation, superficial lymph nodes, skin, eyes, pupils, ears, nose, mouth, teeth, tongue, anus, vagina, penile orifice, mammary glands, testicles, joints, feet, pads, rectal temperature, body condition) and general health observations of dogs in their pen were performed twice daily at least 6 hours apart. The veterinary study investigator assessed all parameters recorded and all clinical findings for their relationship to treatment with fluralaner and/or with the milbemycin oxime plus praziquantel combination product. Body weights were recorded weekly.

## Results and discussion

Throughout the 4-week study period, there were no findings related to the concurrent treatment with fluralaner and milbemycin oxime plus praziquantel (treatment group), or to the treatment with milbemycin oxime plus praziquantel (control group).

Dogs were in the fed status when treated ensuring maximum systemic exposure to fluralaner [5]. Clinical observations were scheduled to cover the period of highest systemic exposure to milbemycin oxime, praziquantel [2,3] and fluralaner [6]. Therefore, clinical signs associated with the concurrent use, e.g. gastrointestinal symptoms, would most likely be apparent at these time points. However, no vomiting, diarrhea, drooling or other clinical signs were observed in any dog during the first hour of clinical observation or during the frequent veterinary examinations performed over the first days following treatment. Occasional clinical findings were observed in individual dogs from the treated and control group during the study (Table 2). Clinical findings included incidences of single small and mild skin lesions (both groups; includes scar, papilloma, sore spots, alopecia, erythema, laceration, graze, scab, scaling), small amounts of serous eye discharge (both groups), excess ear wax (treated group), dental tartar (both groups), penile discharge (control group), transient limping immediately post-treatment (control group), sinus arrhythmia (both groups; single event in the treated group 28 days post-treatment) and loose feces with normal feces (treated group; single event 7 days post-treatment); all of these were minor and transient in both groups, and none affected the general health condition of dogs. The observations in the treated group were considered to be unrelated to the concurrent treatment with fluralaner and milbemycin oxime plus praziquantel or to the use of either product alone, because a similar incidence occurred in the control group, they were already observed pre-treatment and/or the long interval between treatment and observation. All observations were considered to be common findings in a dog colony. The observations in the control group were considered to be unrelated to the treatment with milbemycin oxime plus praziquantel, because they were already observed pre-treatment and/or are common findings in a dog colony.

**Table 2:** **Clinical findings in the treatment and control group post-treatment**

	**Number of dogs affected**	
	Treatment group*	Control group**
**Single small and mild skin lesions (scar/papilloma/sore spots/alopecia/erythema/laceration/graze/scab/scaling)**	4	7
**Small amount of serous eye discharge**	2	1
**Sinus arrhythmia**	1: day 28	(1: day −7)
**Loose feces with normal feces**	1: day 7	0
**Others**	1 excess ear wax, 2 dental tartar	1 penile discharge, 1 dental tartar, 1 transient limping

*None of these observations was considered to be treatment related, because a similar incidence occurred in the control group, they were already observed pre-treatment and/or the long interval between treatment and occurrence. All observations were common findings in a dog colony.

**None of these observations was considered to be treatment related, because they were already observed pre-treatment and/or are common findings in a dog colony.

There were no obvious changes in group mean bodyweights during the study (mean body weights for the treatment group were 21.3 kg pre-treatment and 22.1 kg at study end, and for the control group 21.9 kg pre-treatment and 22.6 kg at study end).

These results are consistent with previous data showing no evidence for interactions of Bravecto™ with other routinely used veterinary medicinal products [3,7].

## Conclusion

Concurrent treatment with fluralaner, milbemycin oxime and praziquantel is well tolerated in dogs.
